# Factors affecting physician decision-making regarding antiplatelet therapy in minor ischemic stroke

**DOI:** 10.3389/fneur.2022.937417

**Published:** 2022-09-01

**Authors:** Tingting Liu, Yanan Li, Xiaoyuan Niu, Yongle Wang, Kaili Zhang, Haimei Fan, Jing Ren, Juan Li, Yalan Fang, Xinyi Li, Xuemei Wu

**Affiliations:** ^1^Department of Neurology, First Hospital of Shanxi Medical University, Taiyuan, China; ^2^Shanxi Medical University, Taiyuan, China; ^3^The Bethune Hospital of Shanxi Province, Taiyuan, China; ^4^General Hospital of Tisco (Sixth Hospital of Shanxi Medical University), Shanxi, China

**Keywords:** factors, physician, decision-making, antiplatelet therapy, minor stroke

## Abstract

**Purpose:**

To identify the most important factors affecting physician decision-making regarding antiplatelet therapy.

**Methods:**

We retrospectively gathered data from minor ischemic stroke patients with NIHSS scores ≤ 5 within 72 h of onset from 2010 to 2018. The population was divided into four groups by initial antiplatelet therapy: aspirin monotherapy (AM), dual antiplatelet therapy with aspirin and a loading dose of clopidogrel (clopidogrel loading dose of 300 mg on the first day; DAPT-ALC), dual antiplatelet therapy with aspirin and no loading dose of clopidogrel (clopidogrel 75 mg daily, no loading dose; DAPT-AUC), and clopidogrel monotherapy (CM).

**Results:**

In total, 1,377 patients were included in the analysis (excluding patients who accepted thrombolytic drugs, participated in other clinical trials, or had not used antiplatelet drugs). The mean ± S.D. age was 62.0 ± 12.7 years; 973 (70.7%) patients were male. The four groups were AM (*n* = 541, 39.3%), DAPT-ALC (*n* = 474, 34.4%), DAPT- AUC (*n* = 301, 21.9%), and CM (*n* = 61, 4.4%). Patients receiving antiplatelet monotherapy were older than those receiving dual antiplatelet therapy (63.7–65.7 vs. 59.6–61.4 years), and the median initial systolic blood pressure level was higher in the DAPT-ALC group than in the other groups (all *P* < 0.05). Patients under 75 years old with an admission SBP lower than 180 mmHg, a history of AM, coronary heart disease, no history of intracerebral hemorrhage, stroke onset occurring after guideline recommendations were updated (the year of 2015), onset-to-arrival time within 24 h, and initial NIHSS score ≤ 3 were more likely to take DAPT-ALC than AM. Compared with DAPT-ALC, DAPT-AUC was associated with an initial SBP level lower than 180 mmHg, a history of smoking, hypertension, no history of ICH, previous treatment with antihypertensives, and onset year after the recommendations were updated.

**Conclusions:**

Many factors affect doctors' decisions regarding antiplatelet therapy, especially guidelines, age, admission SBP level, and hypertensive disease.

## Introduction

Stroke is the leading cause of death and the most burdensome disease in China (1–3). Antiplatelet therapy plays a vital role in the secondary prevention of ischemic stroke (4–6). The CHANCE and POINT trials revealed that early dual antiplatelet therapy could reduce the risk of minor stroke (NIHSS score ≤ 3) (7, 8). However, recent randomized controlled trials (RCTs) have strict population inclusion criteria, and many patients have been excluded (9). In actual clinical practice, there are many cases of super-guided medication, and many patients with NIHSS scores higher than 3 are also treated with dual antiplatelet therapy (10). At the same time, our previous questionnaire survey also found that clinicians pay attention not only to the guideline recommendations but also to whether patients have a risk of disability and make drug decisions according to clinical experience. Therefore, the RCT results do not address all clinical practice needs.

In clinical practice, physician decision-making should follow a rational and algorithmic process in which physicians consciously process the clinical data and apply the most substantial evidence from the findings of clinical trials (11, 12). Physicians need to make complex decisions when treating patients with acute stroke within defined time constraints (13). Any uncertainty is rooted in differences between the patient populations enrolled in clinical trials and “real-world” observational registries and issues of comparability between clinical trials themselves (14). There are even cases of medication use outside the current guidelines (15). Clinicians are concerned about the risk of bleeding, especially cerebral hemorrhage, the most important factor that may require discontinuation of antiplatelet therapy (16). As these variables influence the selection of antiplatelet therapy regimens and may influence the efficacy of such treatments in specific patient populations, it is essential to understand which factors in clinical practice affect a neurologist's selection of an initial antiplatelet therapy regimen in minor ischemic stroke.

## Methods

### Population and study design

This is a multicenter retrospective observational cohort study based on real-world research conducted at three advanced stroke centers in Shanxi Province (First Hospital of Shanxi Medical University, Bethune Hospital of Shanxi Province, Taiyuan Iron and Steel Group Co., Ltd.). The study included patients with acute minor ischemic stroke (NIHSS score ≤ 5) and symptom onset within 72 h. We screened the electronic medical records of all patients hospitalized for minor ischemic stroke during 4 months (March, June, September, and December) in 2010, 2012, 2014, 2016, and 2018.

### Ethics statements

All methods of study were carried out following the relevant guidelines and regulations. Informed consent was waived because of the study's anonymity and minimal risk to the participants. The Ethics Committee of the First Hospital of Shanxi Medical University approved the procedure (No. 2021-K044).

### Data collection

The data were collected from the electronic medical records system by three trained neurologists. Demographic, clinical, imaging, and laboratory data were prospectively collected. Baseline data, including NIHSS scores, were collected for all patients. The stroke subtypes were classified according to the Trial of Org 10172 in Acute Stroke Treatment (TOAST) criteria after complete diagnostic profiling (17). The following data were directly obtained from the registry database: (1) demographics, including age, sex, body mass index, and admission systolic blood pressure and diastolic blood pressure; (2) medical history, including previous transient ischemic attack (TIA), previous stroke, previous coronary artery disease (CAD), previous peripheral artery disease (PAD), hypertension, diabetes mellitus, dyslipidemia, smoking, and atrial fibrillation; (3) medication, including previous antiplatelet medication and previous antihypertensive medication; (4) stroke characteristics and acute treatment, including the time from onset to arrival, initial NIHSS scores, prestroke mRS score, and ischemic stroke subtype according to the TOAST criteria; (5) laboratory data, including white blood cell counts, serum creatinine levels, glucose at presentation, platelet counts, fasting low-density lipoprotein (LDL), and prothrombin time (international normalized ratio); and (6) in-hospital treatment, including statin therapy and initial therapy with antiplatelet drugs.

The study subjects were divided into four groups according to the antiplatelet regimen used during hospitalization: aspirin monotherapy (AM), dual antiplatelet therapy with aspirin and a loading dose of clopidogrel (clopidogrel loading dose of 300 mg on the first day, DAPT-ALC), dual antiplatelet therapy with aspirin and no loading dose of clopidogrel (clopidogrel 75 mg daily, DAPT-AUC) and clopidogrel monotherapy (CM). We divided SBP into <180 and ≥180 mmHg and for additional analysis, into three levels (<140, 140–180, and ≥180 mmHg) that allowed examination of patients at admission.

### Statistical analysis

The frequency (percentage), mean ± S.D., or median (interquartile range, IQR) is reported depending on the variable type. Categorical variables were analyzed using Pearson's chi-squared test or Fisher's exact test, and continuous variables were analyzed using Student's *t*-test or the Wilcoxon rank-sum test, as appropriate. The missing values were substituted with median values for statin treatment. There were two patients with missing age data, 5 (3.34%) patients with missing systolic pressure data, and 6 (4.0%) patients with missing diastolic pressure data. Approximately 26.9–44.3% of the patients had missing laboratory data (such as blood lipids, blood sugar, creatinine, homocysteine, etc.). However, we did not include these data in the final multivariate analysis.

In predefined multiclassification logistic regression analysis, we explored the antiplatelet therapy regimens of interest in patients aged ≥75 or <75 years old; males or females; those with early (≤24 h) or late (24–72 h) arrival; those with an NIHSS score ≤ 3 or an NIHSS score between 4 and 5; systolic blood pressure categorized as lower (<140 mmHg), mild-moderate (140–180 mmHg), or severe (≥180 mmHg); and stroke onset before the updated recommendations (before 2015) or after the updated recommendations (after 2015). Statistical significance was assessed using 95% C.I.s and 2-tailed *P*-values (*P* ≤ 0.05). All analyses were performed with the statistical software packages R (http://www.R-project.org, The R Foundation) and Free Statistics software version 1.3.

## Results

### General characteristics

**Figure 1:**
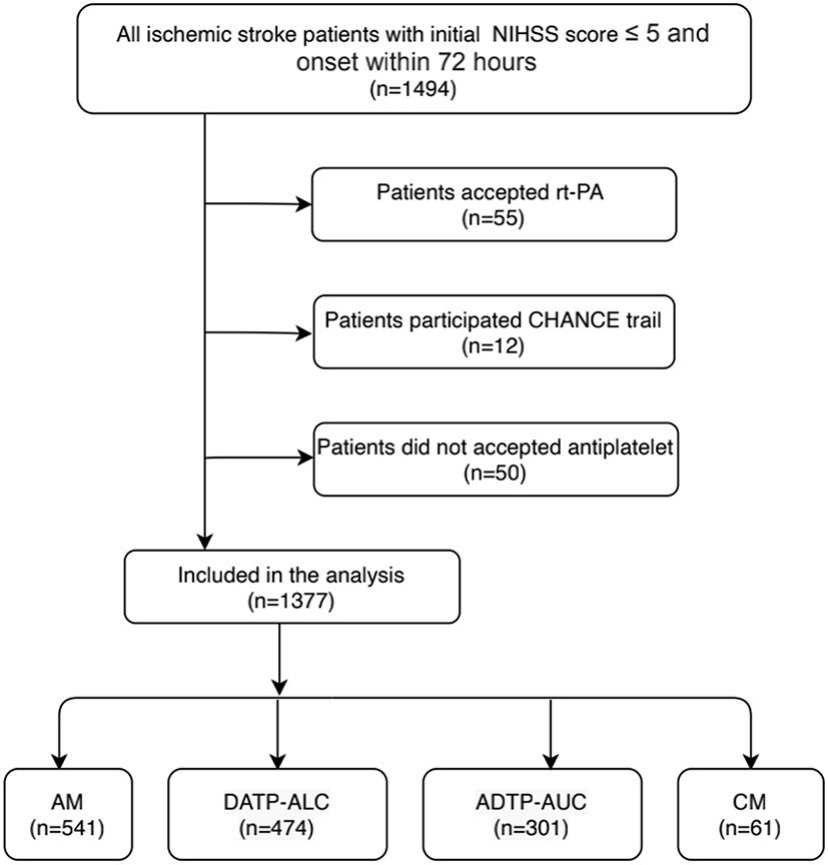
The flow chart of the study population.

Of the 1,494 minor stroke patients who were registered between March 2010 and December 2018, 1,377 patients met the full study eligibility criteria (Figure 1). Of the 1,377 patients with acute minor ischemic stroke, the mean age was 62.0 ± 12.7 years old, and 70.7% were male.

**Figure 2:**
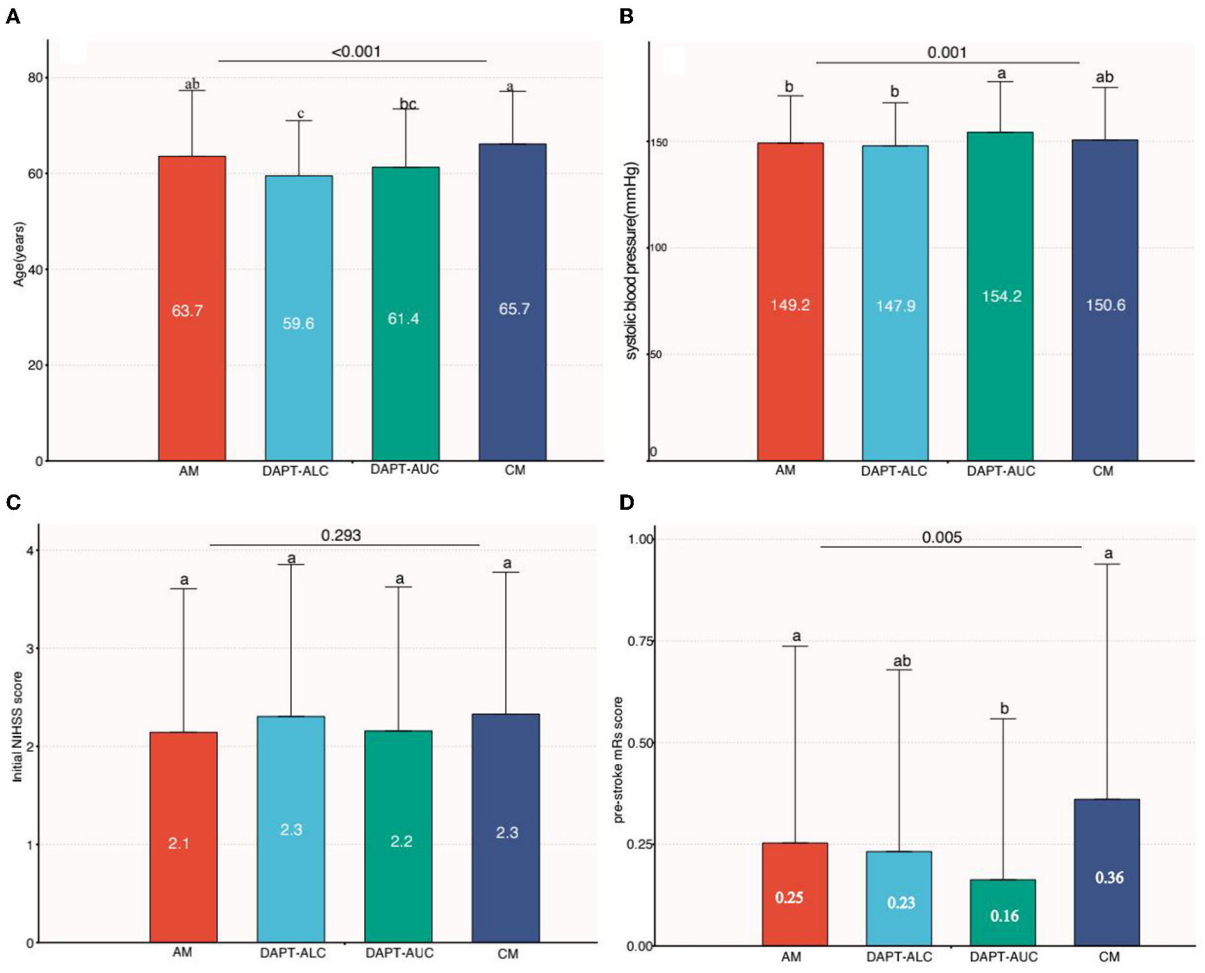
Comparison among groups of continuous variables (age, systolic blood pressure, NIHSS score and pre-stroke mRs score). **(A)** Comparison of age among four groups. **(B)** Comparison of baseline systolic blood pressure among four groups. **(C)** Comparison of baseline NIHSS scores among four groups. **(D)** Comparison of pre-stroke mRs scores among four groups [a and b indicate that when the two groups are compared, if the letters between the two groups are the same, there is no significant difference (e.g., a vs. a, *P* > 0.05), and if the letters between the two groups are different, there is a significant difference between the two groups (e.g., a vs. b, *P* < 0.05)].

The general characteristics of the patients who received aspirin monotherapy (AM, *n* = 541, 39.3%) vs. aspirin plus clopidogrel (administration of a loading dose on the first day) (DAPT-ALC, *n* = 474, 34.4%), aspirin plus clopidogrel (no loading dose on the first day) (DAPT- AUC, *n* = 301, 21.9%) and clopidogrel monotherapy (CM, *n* = 61, 4.4%) are shown in Table 1. The DAPT-ALC group was more likely to be younger; arrive earlier; have a history of smoking, coronary artery disease, and dyslipidemia; have a large artery atherosclerosis stroke etiology; have moderate-to-severe steno-occlusion of the relevant artery; have a lower proportion of ICH and shorter hospitalization; and use statin medication at stroke onset. As shown in Figure 2, compared with that of the monotherapy antiplatelet groups (AM and CM), the median age of the DAPT-AC group was significantly lower (63.7–65.7 vs. 59.6–61.4, *P* < 0.001). The NIHSS score on admission was not different among the four groups, while the median initial SBP level was lower in the DAPT-ALC group. And the median pre-stroke mRs score was lower in the DAPT-ALC group than in the other groups (all *P* < 0.05).

**Table 1:** Comparison of clinical characteristics according to different antiplatelet therapy regimens.

***N* (%)**	**Total**	**AM**	**DAPT-ALC**	**DAPT-AUC**	**CM**	***P*-values**
	***n* = 1,377**	***n* = 541**	***n* = 474**	***n* = 301**	***n* = 61**	
**Clinical items**						
Sex (male)	973 (70.7)	361 (66.7)	349 (73.6)	222 (73.8)	41 (67.2)	0.05
Age, y	62.0 ± 12.7	63.7 ± 13.8	59.6 ± 11.5	61.4 ± 12.0	65.7 ± 11.3	0.000
SBP, mmHg	149.8 ± 22.1	149.2 ± 22.2	147.9 ± 20.2	154.4 ± 23.8	150.5 ± 24.0	0.004
DBP, mmHg	87.6 ± 13.6	86.7 ± 13.3	88.6 ± 13.3	88.3 ± 14.6	85.3 ± 12.0	
**Risk factors and medical history**						
Smoking	600 (43.6)	215 (39.7)	245 (51.7)	120 (39.9)	20 (32.8)	0.000
HTN	812 (59.0)	312 (57.7)	268 (56.5)	195 (64.8)	37 (60.7)	0.12
DM	294 (21.4)	102 (18.9)	114 (24.1)	63 (20.9)	15 (24.6)	0.21
Dyslipidemia	60 (4.4)	23 (4.3)	29 (6.1)	8 (2.7)	0 (0)	0.038
AF	42 (3.1)	14 (2.6)	14 (3.0)	12 (4.0)	2 (3.3)	0.73
TIA	20 (1.5)	8 (1.5)	9 (1.9)	3 (1.0)	0 (0)	0.54
Stroke	323 (23.5)	135 (25)	106 (22.4)	65 (21.6)	17 (27.9)	0.53
CAD	80 (5.8%)	23 (4.3)	35 (7.4)	12 (4.0)	10 (16.4)	0.000
AMI	43 (3.1)	9 (1.7)	17 (3.6)	14 (4.7)	3 (4.9)	0.07
PAD	2 (0.1)	0 (0)	2 (0.4)	0 (0)	0 (0)	0.28
Gastric ulcer	17 (1.2)	6 (1.1)	6 (1.3)	4 (1.3)	1 (1.6)	0.98
ICH	21 (1.5%)	14 (2.6%)	6 (1.3%)	1 (0.3%)	0 (0)	0.044
**Previous treatment**						
Antiplatelet use	127 (9.2)	53 (9.8)	50 (10.5)	19 (6.3)	5 (8.2)	0.23
Antihypertensive use	512 (37.2)	211 (39.0)	182 (38.4)	94 (31.2)	25 (41.0)	0.11
Statin use at admission	494 (35.9)	117 (21.6)	280 (59.1)	81 (26.9)	16 (26.2)	0.000
**Clinical examination**						
TOAST, *n* (%)	0.001					
SVO	651 (45.6)	262 (48.4)	193 (40.7)	152 (50.5)	31 (50.8)	
LAA	555 (38.9)	194 (35.9)	210 (44.3)	116 (38.5)	25 (41)	
C.E.	48 (3.4)	17 (3.1)	8 (1.7)	7 (2.3)	1 (1.6)	
O.E.	89 (6.2)	37 (6.8)	28 (5.9)	14 (4.7)	3 (4.9)	
U.D.	84 (5.9)	31 (5.7)	35 (7.4)	12 (4)	1 (1.6)	
ICAS, *n* (%)	0.001					
No	762 (54.4)	315 (58.8)	242 (51.6)	137 (46.8)	37 (62.7)	
Yes	580 (41.4)	195 (36.4)	222 (47.3)	136 (46.4)	14 (23.7)	
Onset-to-arrival time	0.001					
≤24 h	801 (56.1)	257 (47.5)	299 (63.1)	191 (63.5)	31 (50.8)	
24–72 h	626 (43.9)	284 (52.5)	175 (36.9)	110 (36.5)	30 (49.2)	
Initial NIHSS score	0.44					
≤3	1,064 (76.0)	423 (78.2)	348 (73.4)	230 (76.4)	44 (72.1)	
4–5	343 (24.0)	118 (21.8)	126 (26.6)	71 (23.6)	17 (27.9)	
Hospitalization, days	13.0 (10.0, 15.0)	13.0 (10.0, 14.0)	12.0 (10.0, 14.0)	14.0 (11.0, 15.0)	14.0 (10.0, 15.0)	0.001
**Onset year**						
Before updated recommendations	529 (40.4)	295 (54.5)	106 (22.4)	123 (40.9)	36 (59.0)	
After updated recommendations	782 (59.6)	246 (45.5)	368 (77.6)	178 (59.1)	25 (41)	0.000

Data are shown as n (%) or mean ± S.D.

**Figure 3:**
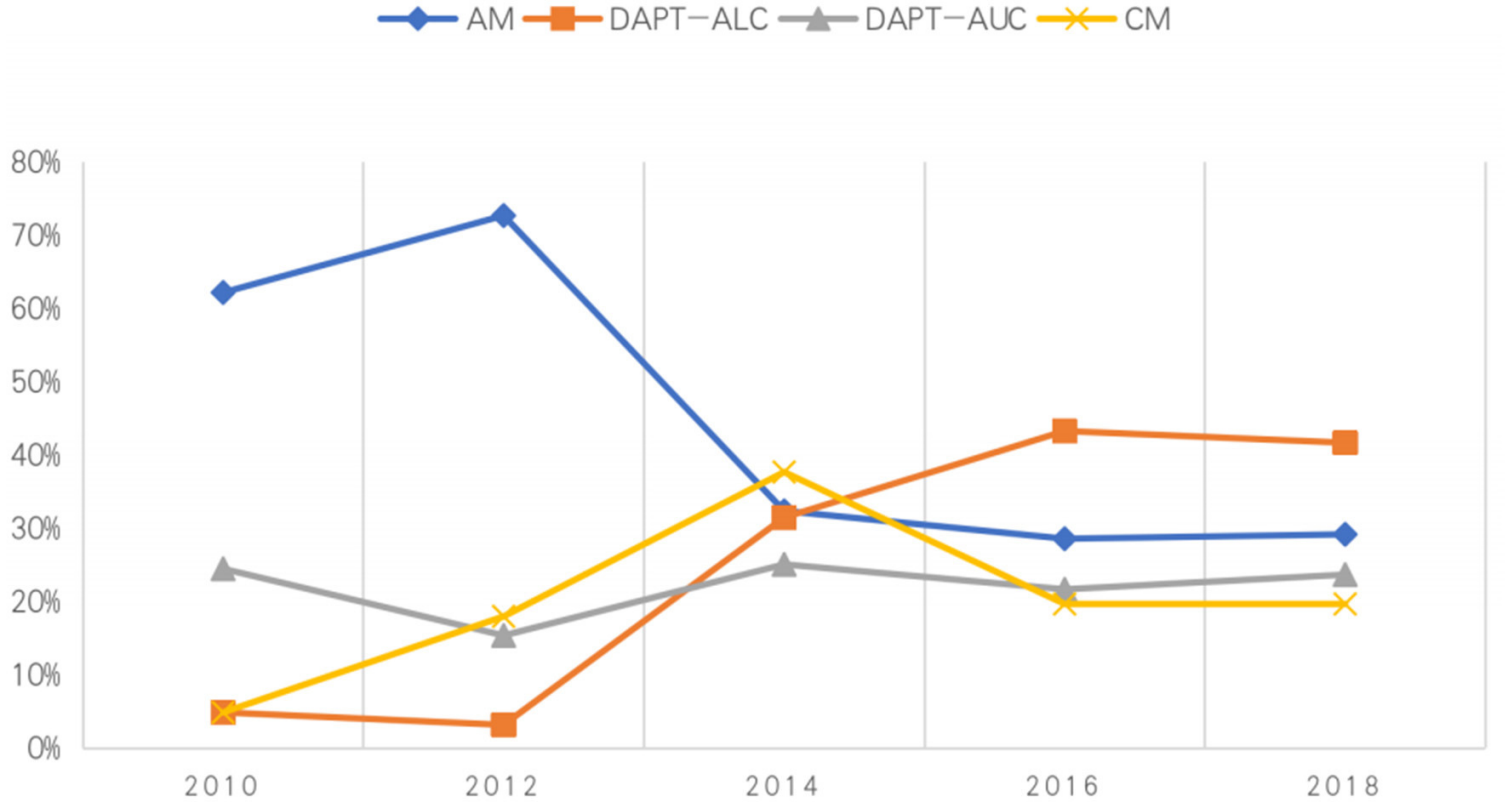
Percentage of patients admitted to the hospital during 2010–2018 who received different medications.

From 2010 to 2018, the proportion of acute minor ischemic stroke patients receiving DAPT-ALC increased yearly, from 4.9 to 41%, while the proportion of those receiving AM decreased from 62.2 to 29.2%. Interestingly, antiplatelet medications were unchanged between 2016 and 2018. Between 2010 and 2018, the proportion of patients receiving DAPT-AUC was almost unchanged (Figure 3).

### Multinomial and multivariate logistic regression analysis

Different drug groups were analyzed by multinomial logistic regression using the AM group as a reference (Table 2). Compared with AM, the choice of DAPT-ALC was associated with the following factors: age under 75 years old, initial SBP lower than 180 mmHg, a history of AM, CHD, no record of ICH, stroke onset occurring after the recommendation update, onset-to-arrival time within 24 h, initial NIHSS score ≤ 3 and use of statin medication at stroke onset. Moreover, the choice of DAPT-AUC was associated with age under 75, initial SBP lower than 180 mmHg, a history of HTN, no history of ICH, previous treatment with antihypertensives, stroke onset after the recommendations were updated, and onset-to-arrival time within 24 h. Compared with DAPT-ALC, the choice of DAPT-AUC was associated with an initial SBP lower than 180 mmHg, a history of smoking, HTN, dyslipidemia, no history of ICH, previous treatment with antihypertensives, onset year after the recommendations were updated, and use of statin medication at stroke onset (Table 3).

**Table 2:** Multinomial logistic regression analysis of different drug groups.

	**DAPT-ALC**		**DAPT-AUC**		**CM**	
***N* (%)**	**OR (95% CI)**	***P*-value**	**OR (95% CI)**	***P*-value**	**OR (95% CI)**	***P*-value**
**Clinical items**						
Sex (male)	1.35 (0.99–1.85)	0.058	1.38 (1.00–1.93)	0.059	1.04 (0.57–1.88)	0.99
**Age group**						
<75	Ref		Ref		Ref	Ref
≥75	0.36 (0.25–0.54)	0.000	0.57 (0.37–0.81)	0.002	0.92 (0.49–1.74)	0.79
**SBP group**						
<140 mmHg	Ref	–	Ref	–	Ref	–
140–180 mmHg	1.00 (0.73–1.36)	0.99	1.00 (0.69–1.37)	0.89	1.04 (0.56–1.91)	0.91
≥180 mmHg	0.59 (0.33–1.04)	0.007	1.46 (0.88–2.42)	0.14	1.53 (0.61–3.83)	0.37
**Risk factors and medical history**						
Smoking	1.31 (0.94–1.83)	0.12	0.76 (0.54–1.07)	0.12	0.66 (0.34–1.26)	0.21
HTN	0.99 (0.68–1.46)	0.98	2.11 (1.44–3.08)	0.000	1.08 (0.52–2.27)	0.83
DM	1.29 (0.92–1.82)	0.15	1.17 (0.81–1.72)	0.4	1.34 (0.69–2.64)	0.39
Dyslipidemia	1.23 (0.65–2.31)	0.53	0.51 (0.22–1.19)	0.12	0	–
AF	1.15 (0.49–2.7)	0.79	1.86 (0.81–4.30)	0.16	0.83 (0.17–4.05)	0.81
TIA	1.46 (0.49–4.30)	0.51	0.86 (0.22–3.43)	0.84	0	–
Stroke	0.80(0.56–1.13)	0.21	0.88 (0.61–1.28)	0.51	1.17 (0.62–2.21)	0.64
CHD	1.93 (1.01–3.68)	0.046	1.02 (0.47–2.21)	0.97	5.16 (2.10–12.7)	0.000
AM	2.30 (0.92–5.71)	0.07	2.97 (1.20–7.36)	0.02	2.38 (0.57–9.92)	0.23
PAD	0	–	0	–	0.73 (0.727–0.73)	0.000.–
ICH	0.24 (0.08–0.75)	0.014	0.10 (0.01–0.80)	0.03	0	–
Gastric ulcer	1.15 (0.31–4.26)	0.83	1.38 (0.35–5.44)	0.66	2.17 (0.25–19.3)	0.49
**Previous treatment**						
Antiplatelet use	0.87 (0.52–1.45)	0.59	0.64 (0.35–1.17)	0.15	0.56 (0.19–1.58)	0.27
Antihypertensive use	0.96 (0.65–1.42)	0.84	0.53 (0.36–0.78)	0.001	0.93 (0.44–1.93)	0.84
**Onset year**						
Before updated recommendations	Ref		Ref		Ref	
After updated recommendations	3.02 (2.21–4.13)	0.000	1.86 (1.35–2.56)	0.000	0.76 (0.42–1.37)	0.36
**Onset-to-arrival time**						
≤24 h	Ref		Ref		Ref	
24–72 h	0.49 (0.37–0.66)	0.000	0.51 (0.38–0.69)	0.000	0.91 (0.52–1.57)	0.73
**Initial NIHSS score group**						
≤3	Ref		Ref		Ref	
4–5	1.39 (1.00–1.93)	0.048	1.2 (0.84–1.71)	0.31	1.20 (0.64–2.23)	0.57
Statin use at admission	3.47 (2.56–4.71)	0.000	1.07 (0.75–1.54)	0.684	1.49 (0.77–2.89)	0.236

^#^Reference is the aspirin monotherapy group.

**Table 3:** Multivariate logistic regression analysis of DAPT-ALC and DAPT-AUC.

	**DAPT-AUC**	
	**OR (95% CI)**	***P*-value**
**Clinical items**		
Sex (Male)	1.33 (0.87–2.04)	0.18
Age group		
<75	Ref	
≥75	1.30 (0.81–2.10)	0.28
**SBP group**		
<140 mmHg	Ref	–
140–180 mmHg	0.93 (0.65–1.35)	0.72
≥180 mmHg	2.51 (1.36–4.63)	0.003
**Risk factors and medical history**		
Smoking	1.83 (1.28–2.68)	0.002
Hypertension	1.97 (1.30–3.00)	0.001
DM	0.94 (0.64–1.40)	0.77
Dyslipidemia	0.36 (0.15–0.87)	0.024
AF	1.32 (0.53–3.32)	0.55
TIA	0.75 (0.18–3.13)	0.55
Stroke	1.12 (0.74–1.71)	0.59
CHD	0.51 (0.23–1.13)	0.09
AM	1.17 (0.51–2.71)	0.71
PAD	–	–
ICH	0.28 (0.03–2.70)	0.27
Gastric ulcer	1.15 (0.29–4.57)	0.84
**Previous treatment**		
Antiplatelet use	0.66 (0.34–1.27)	0.21
Antihypertensive use	0.59 (0.38–0.90)	0.014
**Onset year**		
Before updated recommendations	Ref	
After updated recommendations	0.61 (0.43–0.87)	0.007
**Onset-to-arrival time**		
≤ 24 h	Ref	
24–72 h	1.08 (0.77–1.51)	0.67
**Initial NIHSS score group**		
≤ 3	Ref	
4–5	0.87 (0.60–1.26)	0.46
Statin use at admission	0.32 (0.23–0.45)	0.000

^#^Reference is the DAPT-ALC group.

### The proportions of NIHSS scores × onset-to-arrival times in the different drug groups

The NIHSS score onset-to-arrival time was calculated according to the initial NIHSS score of the grouping. The patients were proportionally divided into four subgroups within each of the different drug groups based on incidence timing. Patients with initial NIHSS scores lower than 3 points and an onset-to-arrival time within 72 h accounted for the largest proportion. The DAPT-ALC group had the highest proportion of patients who met eligibility criteria modeled on the CHANCE trial eligibility criteria, including (1) acute minor ischemic stroke defined as an NIHSS score ≤3 within 24 h of onset (Supplementary Figure S1).

### Outcome events in different drug groups

Supplementary Figure S2 shows the ratio of endpoint events between the different therapy groups. There was no significant difference in the risk of relapse in the hospital with ischemic stroke and TIA among the AM, DAPT-ALC, and DAPT-AUC groups. In contrast, in the CM group, recurrence of TIA was significantly higher than in the other groups. Compared with the DAPT-AC group, the antiplatelet monotherapy groups (AM and CM) had a significantly higher risk of hemorrhagic stroke. However, the hemorrhagic stroke risk in the DAPT-ALC group was higher than that in the DAPT-AUC group (2.1 vs. 1.3%).

## Discussion

This retrospective study of the analysis of medical decisions based on patient data found, for the first time, that patient factors affected doctors' choices of antiplatelet regimens. Approximately two-thirds of the patients were given urgent aspirin plus clopidogrel therapy, and approximately half received DAPT with no loading dose, which is not recommended in the RCTs (7).

In clinical practice, advanced age means more risk factors for cerebrovascular disease (3, 18) and an increased risk of cerebral hemorrhage (19). Our study showed that among patients using aspirin alone, the proportion of patients older than 75 years old was significantly higher than that in the DAPT-ALC and DAPT-AUC groups (9.8 vs. 3.7% and 9.8 vs. 3.6%). Multinomial logistic regression analysis showed that patients under 75 years old were more likely to receive DAPT-ALC [OR = 0.36 (0.25–0.54), *p* < 0.001] or DAPT-AUC [OR = 0.57 (0.37–0.81), *p* = 0.002] than AM. The current bleeding risk score increased for patients older than 75 (20), and patients over 80 were excluded from an RCT (9). However, much research has shown that older age should not be a reason to deny patients antiplatelet therapy (18). Nevertheless, doctors are less likely to select DAPT than monotherapy as the treatment choice for the elderly population (21).

Our study found an interesting phenomenon. In continuous variable analysis, we found that the admission systolic blood pressure level in the DAPT-AUC group was significantly higher than that in the AM, DAPT-ALC, and CM groups (154 vs. 149.2 vs. 150.5 mmHg, *p* < 0.001). Blood pressure readings were divided into three groups, as shown in Supplementary Table S1. The difference in the antiplatelet regimen was more significant when the blood pressure level at admission was ≥180 mmHg. The results showed that when the admission SBP level was ≥180 mmHg, DAPT-AUC therapy was preferred to DAPT-ALC therapy. This may be because high blood pressure means a higher risk of cerebral hemorrhage, but whether antihypertensive treatment is needed in the acute stage of cerebral infarction is still uncertain (22). This may underlie the inconsistent results in recent studies on whether acute blood pressure needs to decrease during the acute period (23, 24).

The analysis of stroke risk factors and previous disease history showed that there were significant differences in the smoking rate and CAD among the different treatment groups. People with previous bleeding events are excluded from the population in RCTs, but there are guidelines that recommend new antiplatelet therapy after bleeding events (4, 25). Analysis of the history of bleeding disease showed that there was no difference in the history of digestive tract ulcers among the treatment groups but that there was a slightly significant difference in the history of cerebral hemorrhage (*p* = 0.044), which may be related to the number of samples. Multiple regression analysis showed that patients with a previous history of cerebral hemorrhage were more likely to be given single antiplatelet therapy, while a history of gastrointestinal hemorrhage did not affect the choice of antiplatelet therapy. In recent years, some studies have reported a higher ischemic and bleeding risk in combination with peripheral diseases and other vascular events (26).

Clinicians are more likely to make decisions based on the latest guidelines. After the revision of the guidelines, the proportion of DAPT-ALC selection increased from 18.9 to 43.6%, the proportion of AM use decreased from 52.6 to 29.1%, and that of DAPT-AUC selection changed from 21.6 to 21.1%. Regardless of how the guidelines are revised, some patients are still given DAPT-AUC. Multiple regression analysis showed that the recommendations of the guidelines had an effect on both DAPT with a loading dose and DAPT without a loading dose, with OR values of 3.05 (2.233–4.17) and 1.85 (1.35–2.55), respectively. The guidelines are still the best evidence to guide treatment, but actual clinical patients are different from the typical patients included in RCTs, so treatments outside of those recommended by the guidelines are still used in clinical practice. In particular, some studies showed that patients could benefit from changing to a new antiplatelet drug or adding and additional one upon onset of a new stroke (27).

Multiple logistic regression showed that patients with within 24 h of onset might be given DAPT-ALC therapy, regardless of whether their NIHSS score is ≤ 3 or between 4 and 5 points. This may be because the guidelines specify that DAPT therapy should be administered within 24 h of onset, but there is no uniform standard for the concept of minor stroke (5). In Supplementary Figure S1, the distribution is presented according to the treatment plan and initial NIHSS score × onset-to-arrival time. There was no significant difference in the distribution of initial NIHSS score × onset-to-arrival time among the different treatment groups. The analysis of the incidence of primary events among the treatment groups revealed no significant difference in ischemic stroke and TIA among the three groups, except in the CM group. The incidence of in-hospital TIA in the clopidogrel group was significantly higher than that in the other three groups. There was an interesting phenomenon in the incidence of cerebral hemorrhage: the incidence was higher in the single antiplatelet group than in the DAPT group, which may be related to the higher risk of bleeding among patients who choose to be treated with antiplatelet monotherapy. The incidence of cerebral hemorrhage in the loading dose group was significantly higher than that in the group without a loading dose. This may reflect the complexity and uncertainty of clinical decision-making (28) and a higher risk of ischemic and bleeding incidence in the real world (29). However, a larger sample of data is needed to confirm this hypothesis.

### Limitations and shortcomings

First, this is a retrospective study based on clinical patient data, and thus, the factors of medical decision-making, such as the impact of the medical environment and doctors' professional level on decision-making, could not be fully analyzed. Second, there may be a deviation in retrospective studies due to incomplete data records. Third, our sample size was small, and some results may be due to chance, which still needs further confirmation.

## Conclusion

Many factors affect the decision-making of doctors regarding antiplatelet therapy, especially guidelines, the patient's age, the admission SBP level, and hypertensive disease. However, there is still a need for an analysis of data from a large sample to investigate the impact of decisions on short- and long-term prognosis.

## Data availability statement

The original contributions presented in the study are included in the article/Supplementary material, further inquiries can be directed to the corresponding author/s.

## Ethics statement

The studies involving human participants were reviewed and approved by the Ethics Committee of the First Hospital of Shanxi Medical University (No. 2021-K044). Written informed consent for participation was not required for this study in accordance with the national legislation and the institutional requirements.

## Author contributions

TL and YL wrote the manuscript. YW analyzed the data. KZ, HF, JR, JL, and YF performed the research and collected data. XL and XW performed supervision for other centers. XN conceived, designed, and supervised the study. All authors reviewed and finally approved the manuscript.

## Conflict of interest

The authors declare that the research was conducted in the absence of any commercial or financial relationships that could be construed as a potential conflict of interest.

## Publisher's note

All claims expressed in this article are solely those of the authors and do not necessarily represent those of their affiliated organizations, or those of the publisher, the editors and the reviewers. Any product that may be evaluated in this article, or claim that may be made by its manufacturer, is not guaranteed or endorsed by the publisher.

## Abbreviations

SBP: systolic blood pressure
DBP: diastolic blood pressure
HTN: hypertension
DM: diabetes mellitus
AF: atrial fibrillation
TIA: transient ischemic attack
CAD: coronary artery disease
AMI: acute myocardial infarction
PAD: peripheral artery disease
ICH: intracranial hemorrhage
ICAS: intracranial cerebral atherosclerosis
TOAST: Trial of ORG 10172 in Acute Stroke Treatment
LAA: large artery atherosclerosis
SVO: small vessel occlusion
CE: cardioembolic
OE: other etiology
UD: undetermined etiology
mRS: modified Rankin Scale
NIHSS: National Institutes of Health Stroke Scale
Before updated recommendations: before 2015
After updated recommendations: after 2015
AM: aspirin monotherapy
CM: clopidogrel monotherapy
DAPT-ALC: dual antiplatelet therapy with aspirin and a loading dose of clopidogrel (clopidogrel loading dose of 300 mg on the first day)
DAPT-AUC: dual antiplatelet therapy with aspirin and no loading dose of clopidogrel (clopidogrel 75 mg daily)
DAPT-AC: dual antiplatelet therapy with aspirin and clopidogrel
CHANCE: The Clopidogrel in High-risk patients with Acute Nondisabling Cerebrovascular Events
POINT: Platelet-Oriented Inhibition in New TIA and Minor Ischemic Stroke.
