# Corrigendum: Factors affecting physician decision-making regarding antiplatelet therapy in minor ischemic stroke

**DOI:** 10.3389/fneur.2022.1053112

**Published:** 2022-10-14

**Authors:** Tingting Liu, Yanan Li, Xiaoyuan Niu, Yongle Wang, Kaili Zhang, Haimei Fan, Jing Ren, Juan Li, Yalan Fang, Xinyi Li, Xuemei Wu

**Affiliations:** ^1^Department of Neurology, First Hospital of Shanxi Medical University, Taiyuan, China; ^2^Shanxi Medical University, Taiyuan, China; ^3^The Bethune Hospital of Shanxi Province, Taiyuan, China; ^4^General Hospital of Tisco (Sixth Hospital of Shanxi Medical University), Shanxi, China

**Keywords:** factors, physician, decision-making, antiplatelet therapy, minor stroke

In the published article, there was an error in affiliations 1 and 2. Instead of “1 Shanxi Medical University, Taiyuan, China,” it should be “2 Shanxi Medical University, Taiyuan, China.” Instead of “2 Department of Neurology, First Hospital of Shanxi Medical University, Taiyuan, China,” it should be “1 Department of Neurology, First Hospital of Shanxi Medical University, Taiyuan, China.”

The authors apologize for this error and state that this does not change the scientific conclusions of the article in any way. The original article has been updated.

## Publisher's note

All claims expressed in this article are solely those of the authors and do not necessarily represent those of their affiliated organizations, or those of the publisher, the editors and the reviewers. Any product that may be evaluated in this article, or claim that may be made by its manufacturer, is not guaranteed or endorsed by the publisher.

