# Supplementary figures and images for: Factors affecting physician decision-making regarding antiplatelet therapy in minor ischemic stroke
Source: Front Neurol. 2022 Sep 1;13:937417. doi: 10.3389/fneur.2022.937417 (PMC9477012; doi:10.3389/fneur.2022.937417)

Figure S1 The proportion of NIHSS score  $\times$  onset to arrival time in different drug groups

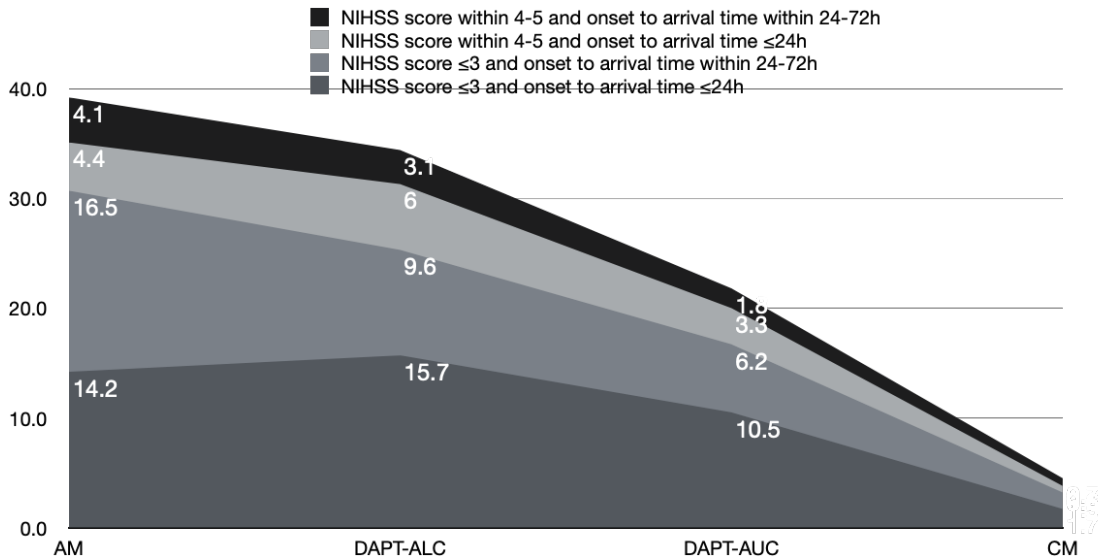

Supplement: Supplementary file 1 [file Data_Sheet_1.PDF]

Figure S2 The outcome events in different drug groups

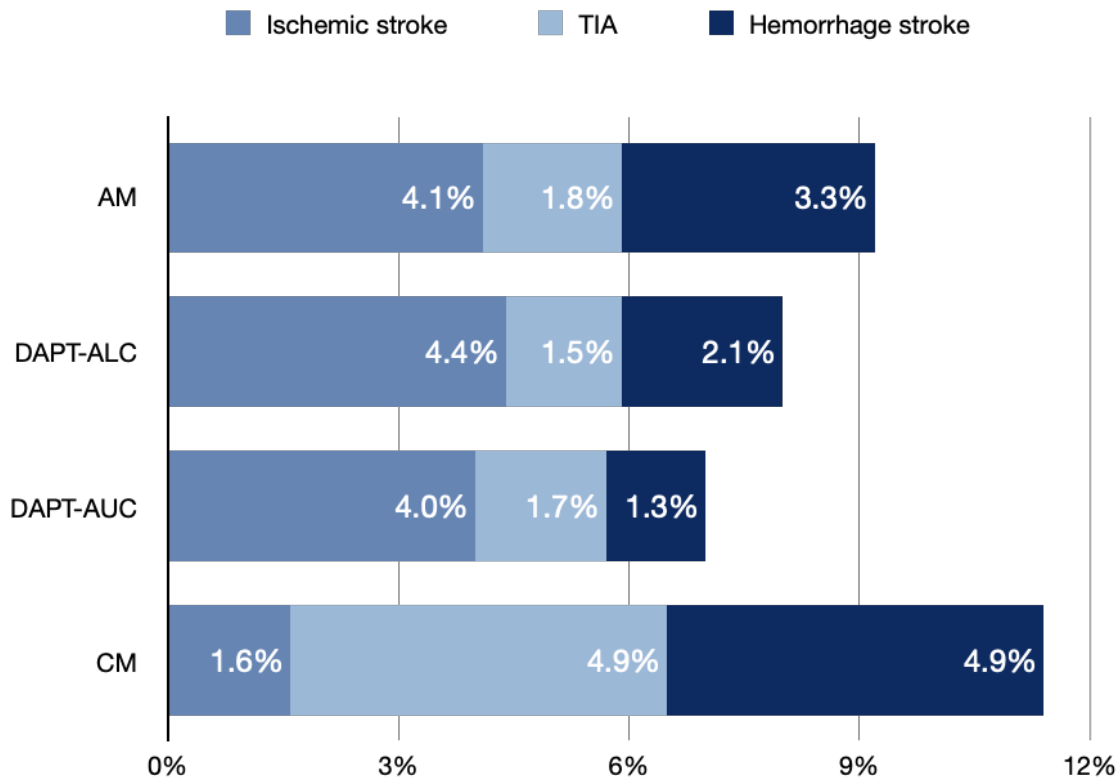

Supplement: Supplementary file 2 [file Data_Sheet_2.PDF]
