# Supplementary material for: Factors affecting physician decision-making regarding antiplatelet therapy in minor ischemic stroke
Source: Front Neurol. 2022 Sep 1;13:937417. doi: 10.3389/fneur.2022.937417 (PMC9477012; doi:10.3389/fneur.2022.937417)
Supplement: Supplementary file 3 [file Table_1.docx]

| Table S1 **Comparison of clinical characteristics according to sex** | | | | |
| --- | --- | --- | --- | --- |
| Variables | Total (n=1377) | 0 (n=404) | 1 (n=973) | P |
| Hypertension, n (%) | 812 (59.0) | 274 (67.8) | 538 (55.3) | <0.001 |
| Diabetes, n (%) | 294 (21.4) | 110 (27.2) | 184 (18.9) | <0.001 |
| Dyslipidemia, n (%) | 60 (4.4) | 13 (3.2) | 47 (4.8) | 0.234 |
| ICH, n (%) | 21 (1.5) | 8 (2) | 13 (1.3) | 0.518 |
| Onset to arrival time, n (%) |  |  |  | 0.492 |
| ≤24 h | 778 (56.5) | 222 (55) | 556 (57.1) |  |
| 24-72 h | 599 (43.5) | 182 (45) | 417 (42.9) |  |
| Initial NIHSS score, n (%) |  |  |  | 0.88 |
| ≤3 | 1045 (75.9) | 305 (75.5) | 740 (76.1) |  |
| 4-5 | 332 (24.1) | 99 (24.5) | 233 (23.9) |  |
| AF, n (%) | 42 (3.1) | 19 (4.7) | 23 (2.4) | 0.033 |
| TIA, n (%) | 20 (1.5) | 9 (2.2) | 11 (1.1) | 0.193 |
| Antiplatelet use, n (%) | 127 (9.2) | 43 (10.6) | 84 (8.6) | 0.284 |
| **Antihypertensive use**, n (%) | 512 (37.2) | 191 (47.3) | 321 (33) | < 0.001 |
| Antiplatelet drugs admission, n (%) |  |  |  | 0.051 |
| AM | 541 (39.3) | 180 (44.6) | 361 (37.1) |  |
| DAPT-ALC | 474 (34.4) | 125 (30.9) | 349 (35.9) |  |
| DAPT-AUC | 301 (21.9) | 79 (19.6) | 222 (22.8) |  |
| CM | 61 (4.4) | 20 (5) | 41 (4.2) |  |
| Stroke, n (%) | 323 (23.5) | 93 (23) | 230 (23.6) | 0.86 |
| CAD, n (%) | 80 (5.8) | 29 (7.2) | 51 (5.2) | 0.203 |
| AMI, n (%) | 43 (3.1) | 8 (2) | 35 (3.6) | 0.161 |
| PAD, n (%) | 2 (0.1) | 1 (0.2) | 1 (0.1) | 0.501 |
| Abbreviations: AF: atrial fibrillation; TIA: transient ischemic attack; CAD: coronary artery disease; AMI: acute myocardial infarction; PAD: peripheral artery disease; ICH: intracranial hemorrhage; NIHSS: National Institutes of Health Stroke Scale; AM: aspirin monotherapy; CM: clopidogrel monotherapy; DAPT-ALC: dual antiplatelet therapy with aspirin and a loading dose of clopidogrel (clopidogrel loading dose of 300 mg on the first day); DAPT-AUC: dual antiplatelet therapy with aspirin and no loading dose of clopidogrel (clopidogrel 75 mg daily); Data are shown as n (%). | | | | |
